# Characterization of intracortical synaptic connections in the mouse anterior cingulate cortex using dual patch clamp recording

**DOI:** 10.1186/1756-6606-2-32

**Published:** 2009-10-15

**Authors:** Long-Jun Wu, Xiangyao Li, Tao Chen, Ming Ren, Min Zhuo

**Affiliations:** 1 Department of Physiology, Faculty of Medicine, University of Toronto center for the Study of Pain, University of Toronto, 1 King's College Circle, Toronto, Ontario M5S 1A8, Canada; 2 Department of Brain and Cognitive Sciences, Seoul National University, Seoul 151-746, Korea

## Abstract

The anterior cingulate cortex (ACC) is involved in sensory, cognitive, and executive functions. Studies of synaptic transmission and plasticity in the ACC provide an understanding of basic cellular and molecular mechanisms for brain functions. Previous anatomic studies suggest complex local interactions among neurons within the ACC. However, there is a lack of functional studies of such synaptic connections between ACC neurons. In the present study, we characterized the neuronal connections in the superficial layers (I-III) of the mouse ACC using dual whole-cell patch clamp recording technique. Four types of synaptic connections were observed, which are from a pyramidal neuron to a pyramidal neuron, from a pyramidal neuron to an interneuron, from an interneuron to a pyramidal neuron and from an interneuron to an interneuron. These connections exist among neurons in layer II/III or between neurons located layer I and II/III, respectively. Moreover, reciprocal connections exist in all four types of paired neurons. Our results provide the first key evidence of functional excitatory and inhibitory connections in the ACC.

## Introduction

The anterior cingulate cortex (ACC) is the frontal part of the cingulate cortex, which forms a large region around the rostrum of the corpus callosum in the mammalian brain. Studies from both animals and humans consistently demonstrate that the ACC plays a critical role in emotional and attentive responses to internal and external stimulation, such as pain, fear, anxiety, sexual arousal, learning and memory [1-9]. For example, electric or chemical activation of the ACC facilitates the spinal nociceptive tail-flick reflex [10], induces fear memory [11] and aversive learning [12]. Furthermore, peripheral stimulation activates immediate early genes as well as long-term plastic changes in the ACC [13-16]. Therefore, synaptic transmission and plasticity in the ACC are important for ACC-related brain functions.

The ACC is a part of the thalamo-limbic-cortical circuitry where it receives various sensory inputs from the thalamus and sends outputs to motor cortex as well as several subcortical brain regions such as the hippocampus, amygdala and hypothalamus [7,17]. Anatomically, the ACC itself contains several layers including layer I, II, III, V and VI. Layer I contains small local interneurons. However, many projecting fibers from other central nuclei end or pass through layer I. Neurons in layers II-III are mainly pyramidal cells, which receive sensory inputs from the medial thalamus and send projections to deep layers. Pyramidal neurons in layer V receive input from layers II-III as well as the thalamus and project to cortical and subcortical structures [7,18-20]. In layers II-VI, there are also many local interneurons. It has been proposed that neurons in the ACC may form local excitatory and inhibitory connections. However, direct evidence for functional connections between pyramidal neurons and/or interneurons within the ACC has not been reported.

Our previous studies indicate that fast excitatory synaptic is mediated by glutamate [14,21] and inhibitory synaptic transmission is mainly mediated by GABA transmission in the ACC [22]. However, the stimulations used in these studies cannot differentiate the exact afferent inputs to recorded neurons, which could be derived from either intra-ACC or from subcortical areas. In the present study, we have examined direct neuronal connections using dual whole-cell patch clamp recording method in ACC slices. To our knowledge, this is the first study using dual recording technique in the ACC region. Our results show different pairs of uni- and bi-directional synaptic connections between pyramidal neurons and/or interneurons in the ACC.

## Methods

### Animals

All C57BL/6 mice were purchased from Charles River and were maintained on a 12 h light/dark cycle with food and water provided *ad libitum*. Experiments were performed on 3-4 weeks old mice. The Animal Studies Committee at the University of Toronto approved all experimental protocols.

### Brain slice preparation

Mice were deeply anesthetized with isoflurane. Coronal brain slices (300 μm) containing the ACC were prepared using standard methods [14,22,23]. Slices were transferred to a submerged recovery chamber with oxygenated (95% O_2 _and 5% CO_2_) artificial cerebrospinal fluid (ACSF) containing (in mM): 124 NaCl, 2.5 KCl, 2 CaCl_2_, 2 MgSO_4_, 25 NaHCO_3_, 1 NaH_2_PO_4_, 10 glucose at room temperature for at least one hour.

### Dual whole-cell patch clamp recordings in ACC slices

After one-hour recovery, slices were placed in a recording chamber on the stage of an Olympus BX51WI microscope (Tokyo, Japan) with infrared DIC optics for visualization of whole-cell patch clamp recordings. Neurons were recorded from layer I or II/III with an Axon 200B amplifier (Molecular devices, CA). Three types of intracellular solutions were used: (1) normal intracellular solution (in mM): K-gluconate, 120; NaCl, 5; MgCl_2 _1; EGTA, 0.5; Mg-ATP, 2; Na_3_GTP, 0.1; HEPES, 10; pH 7.2; 280-300 mOsmol, (2) high Cl^- ^intracellular solution: same as normal intracellular solution except K-gluconate (120 mM) was replaced by KCl (60 mM) and K-gluconate (60 mM), and (3) low Cl^- ^intracellular solution: same as normal intracellular solution except K-gluconate (120 mM) was replaced by Cs-MeSO_3 _(120 mM). The Cs-MeSO_3 _was used to improve the clamp quality. The membrane potential was held at -70 mV for postsynaptic neurons to record unitary excitatory postsynaptic current (uEPSCs) with the normal intracellular solution, while held at 0 mV to record outward unitary inhibitory postsynaptic currents (uIPSCs) with the low Cl^- ^intracellular solution and at -70 mV to record inward uIPSCs with the high Cl^- ^intracellular solution. Access resistance was 15-30 MΩ and was monitored throughout the experiment. In dual whole-cell recording, action potentials were elicited by applying brief (1 ms) depolarizing current pulses (200 pA) under current clamp configuration or applying brief (1 ms) depolarizing voltage pulse (from -70 mV to +20 mV) at 0.1 Hz. The latency of postsynaptic currents was determined by the time difference between the peak of presynaptic spikes and the onset of postsynaptic current. The rise time of postsynaptic currents is the time from 10% to 90% of maximal peak current, while the decay time is the time from maximal peak amplitude to 37% of the peak amplitude. Half-width of postsynaptic currents is the width (duration) at half-maximal peak amplitude.

### Biocytin labeling and confocal imaging

After recording, brain slices were immediately fixed in 4% paraformaldehyde in 0.1 M phosphate buffer (PB, pH 7.4) for 1 hr at room temperature. Slices were then transferred to 0.01 M phosphate buffer saline (PBS, pH 7.4) containing 1% Triton X-100 (PBS-triton) and stored at 4°C for 48 hr. After this, sections were rinsed with 3% hydrogen peroxide in 0.01 M PBS for 30 min. After thoroughly washing with PBS, the tissue was incubated with Fluorescein (DTAF) Streptavidin (016-010-084, 1:200 dilution, Jackson) containing 3% fish gelatin (Sigma) in PBS-Triton for 4 hours at room temperature. The immunofluorescence-labeled sections were then rinsed in PBS, mounted onto glass slides, air dried, cover-slipped with a mixture of 50% (v/v) glycerin and 2.5% (w/v) triethylene diamine in 0.01 M PBS, and observed with an confocal microscope (FV-1000; Olympus, Tokyo, Japan) under appropriate filter for DTAF (excitation 492 nm; emission 520 nm).

### Data analysis and statistics

Results were analyzed by t-test and paired t-test where necessary. All data are expressed as mean ± S.E.M. In all cases, *P *< 0.05 was considered statistically significant.

## Results

**Figure 1:**
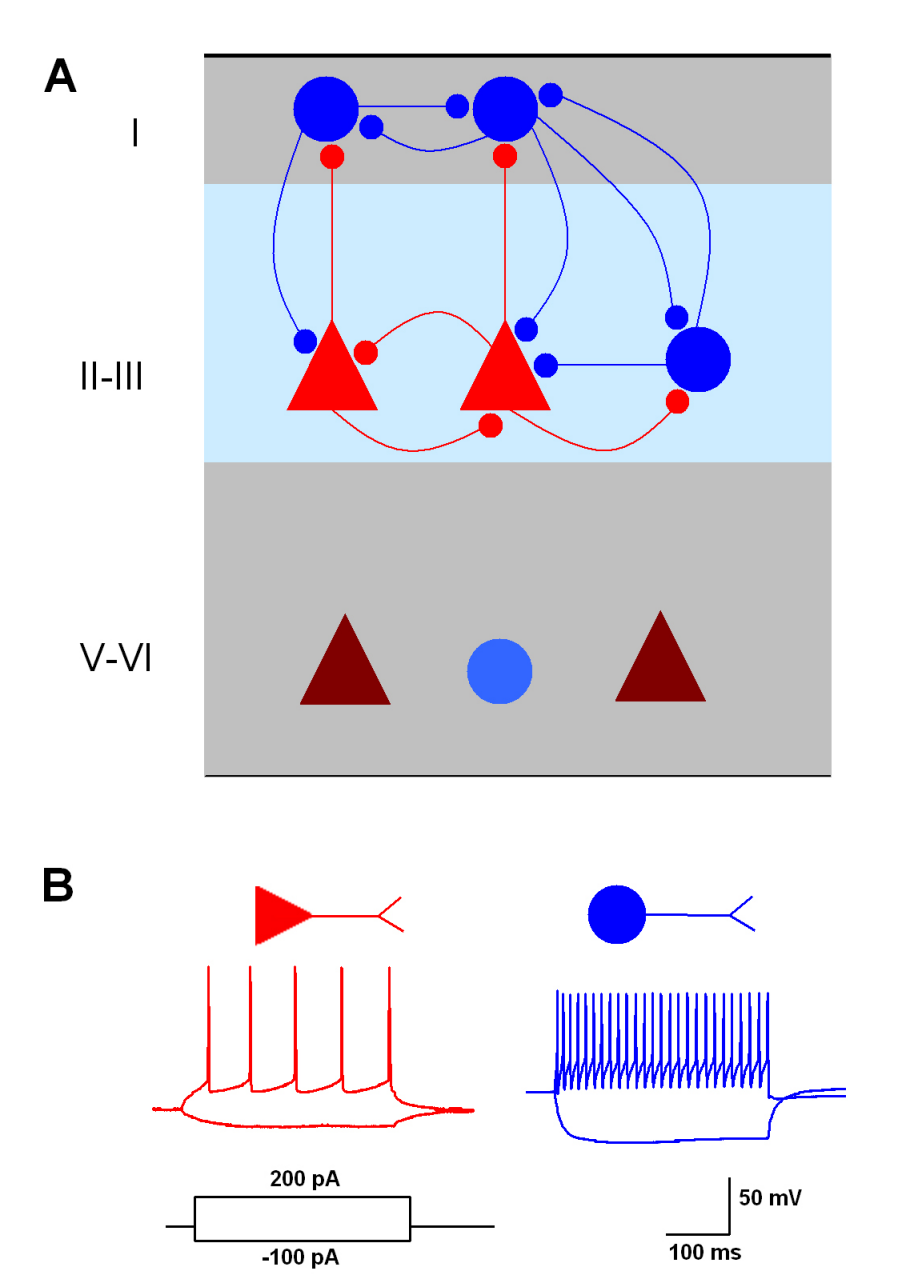
**Diagram for the proposed synaptic connections in the ACC circuit**. ***(A) ***A simplified diagram illustrates the possible synaptic connections in the ACC. Pyramidal neurons are indicated as red triangles, while interneurons are blue circle. ***(B) ***Typical firing patterns for pyramidal neurons (left) and interneurons (right) after current injection of 200 pA for 400 ms. The resting membrane potentials for the pyramidal neuron and interneuron are --71.3 mV and --67.8 mV, respectively.

We proposed that there are complex synaptic connections in the ACC (Figure 1A). In the superficial layers such as I and II/III, pyramidal neurons may send projections to pyramidal neurons and interneurons, interneurons may target pyramidal neurons and interneurons, and reciprocal connections may exist between two pyramidal neurons, a pyramidal neuron and an interneuron, or two interneurons. To test the possibilities, dual recordings were performed in neuronal pairs in layers I and II/III of the ACC. Although we have found that pyramidal neurons and interneurons could also be divided into several subtypes based on firing patterns [24] (Cao et al., unpublished data), we only classify these neurons into pyramidal neuron (Py) or interneuron (In) to simplify the possibilities of neuronal connections. The typical firing patterns for pyramidal neurons and fast-spiking interneurons are shown in Figure 1B after current injection (200 pA, 400 ms).

Dual whole-cell patch clamp recordings were performed in the layer I and II/III neurons in the ACC from juvenile, wild-type mice (3-4 week old). This study included recordings from 49 mice and a total of 71 successful synaptic connections were obtained from around 223 pairs of neurons recorded (about 31% success rate). All pyramidal neurons were recorded from layer II/III while interneurons were recorded from layer I or II/III as specified in the results. Pyramidal neurons and interneurons were distinguished based on their morphology (Figure 5), membrane properties, single action potential shape (Table 1), and firing pattern (Figure 1B).

**Table 1:** Membrane properties and action potential parameters in pyramidal neurons and interneurons in the superficial layers of the ACC

	**Pyramidal neuron**	**Interneuron**	**Significant Difference**
Number of neurons tested	15	15	
Membrane capacitance, pF	161.5 ± 5.7	44.2 ± 3.0	P < 0.001
Input resistance, MΩ	206.3 ± 24.9	297.2 ± 30.62	P < 0.05
Membrane tau, ms	4.0 ± 0.2	1.0 ± 0.1	P < 0.001
Resting membrane potential, mV	-72.9 ± 1.7	-70.2 ± 1.5	P = 0.23
Action potential threshold, mV	-43.4 ± 1.2	-44.7 ± 0.7	P = 0.33
Action potential amplitude, mV	92.0 ± 1.2	65.1 ± 2.8	P < 0.001
Action potential half-width, ms	1.3 ± 0.1	0.79 ± 0.04	P < 0.001

### Excitatory connections between ACC neurons

**Figure 2:**
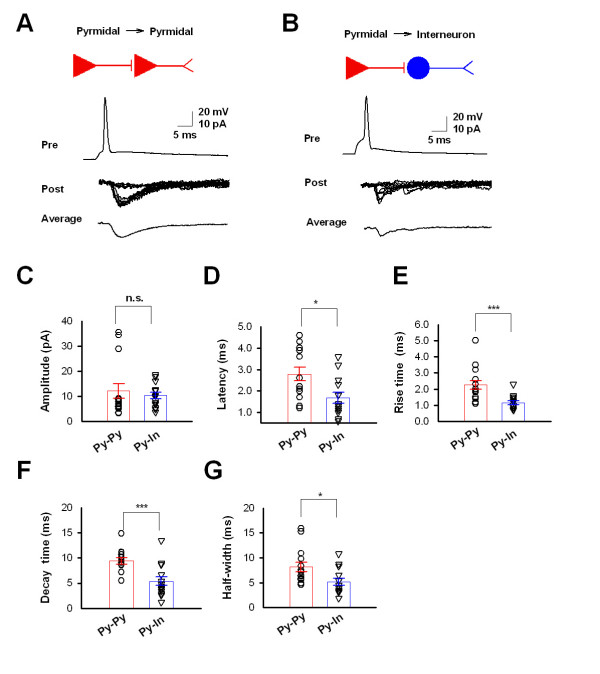
**Excitatory synaptic transmission in the ACC**. **(*A*) **Diagram and sample traces of synaptic connections from pyramidal neurons to pyramidal neurons. Pre, action potential is induced in the presynaptic pyramidal neuron under current clamp. Post, inward uEPSCs with failure are recorded in the postsynaptic neuron under voltage clamp holding at --70 mV. Average, the trace of the average of 10 original traces. **(*B*) **Diagram and sample traces of synaptic connections from pyramidal neurons to interneurons. **(*C*) **Pooled data (open circle and triangle) and summarized results (bar graph) showing the amplitude of uEPSCs for Py-Py and Py-In. n.s., no significance. **(*D-G*) **Pooled data (open circle and triangle) and summarized results (bar graph) indicate the latency ***(D)***, rise time ***(E)***, decay time ***(F) ***and half-width ***(G) ***of uEPSCs for Py-Py and Py-In. Unitary EPSCs from Py-In pairs have faster kinetics than those from Py-Py pairs. * P < 0.05, ** P < 0.01, *** P < 0.001.

To study excitatory synaptic transmission in the ACC, we recorded synaptic transmissions between two pyramidal neurons (Py-Py) or from a pyramidal neuron to an interneuron (Py-In). Presynaptic neurons were recorded under current clamp and action potentials were induced by current injection. The postsynaptic neurons were recorded under voltage clamp holding at -70 mV. Successful recordings were obtained in 14 pairs of two pyramidal neurons in layer II/III. When a single action potential was evoked in the presynaptic neuron, monosynaptic inward currents, which are putatively glutamatergic, were obtained in postsynaptic neurons with amplitude of 12.1 ± 3.0 pA and a failure rate of 19.0 ± 6.1% (n = 14) (Figure 2A and 2C). When we recorded the pair of pyramidal neuron to interneurons, 13 pairs exhibited glutamatergic responses with of 10.4 ± 1.3 pA and a failure rate of 31.5 ± 8.4% (n = 13) (Figure 2B and 2C). Among them, 4 interneurons were located in layer I and another 9 neurons were located in layer II/III. Since there is no obvious difference uEPSCs between these two groups of interneurons, data were pooled together. These results indicate that excitatory connections exist between pyramidal neurons in layer II/III and pyramidal neuron to interneurons in both layer I and layer II/III.

Next, we compared the latency and kinetics of uEPSCs between pairs of Py-Py and Py-In. We found that the uEPSCs to interneurons (Py-In) have significant shorter latency than those to pyramidal neurons (Py-Py) (Py-Py, 2.8 ± 0.3, n = 14; Py-In, 1.7 ± 0.3, n = 13; P < 0.05) (Figure 2D). Moreover, the kinetics of uEPSC to interneurons is dramatically faster than that to pyramidal neurons, showing shorter rise time, decay time and half-width (Figure 2E-G).

### Inhibitory connections between ACC neurons

**Figure 3:**
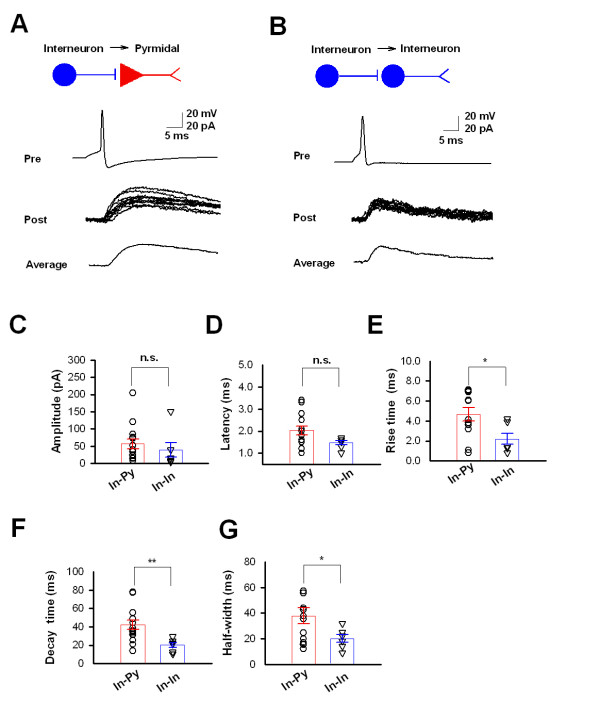
**Inhibitory synaptic transmission in the ACC**. **(*A*) **Diagram and sample traces of synaptic connections from interneurons to pyramidal neurons. Pre, action potential is induced in the presynaptic interneuron under current clamp. Post, outward uIPSCs is recorded in postsynaptic neuron under voltage clamp holding at 0 mV. Average, the trace of the average of 10 original traces. **(*B*) **Diagram and sample traces of synaptic connections from interneuron to interneuron. **(*C*) **Pooled data (open circle and triangle) and summarized results (bar graph) showing the amplitude of uIPSCs for In-Py and In-In. **(*D-G*) **Pooled data (open circle and triangle) and summarized results (bar graph) indicate the latency **(*D*)**, rise time **(*E*)**, decay time **(*F*) **and half-width **(*G*) **of uEPSCs for In-Py and In-In. Unitary IPSCs from In-In pairs have faster kinetics than those from In-Py pairs.

We then studied inhibitory synaptic transmissions from an interneuron to a pyramidal neuron (In-Py) or between two interneurons (In-In). Presynaptic interneurons were recorded under current clamp while postsynaptic pyramidal neurons or interneurons were under voltage-clamp holding at 0 mV. Therefore, the putatively GABAergic outward current were obtained in postsynaptic neurons. We successfully recorded 14 functional pairs of In-Py connections, in which uIPSCs showed amplitude of 56.4 ± 14.4 pA and a failure rate of 3.9 ± 2.7% (n = 14) (Figure 3A and 3C). Among them, 6 interneurons were recorded in the layer I while the other 8 interneurons were found in layers II/III. For In-In connections, 8 functional pairs were recorded. The amplitude of uIPSCs was 38.8 ± 20.9 pA and the failure rate was 18.3 ± 10.7% (n = 7) (Figure 3B and 3C). Among 8 functional In-In connections, 2 paired neurons were both in layer I and another 2 paired neurons were both in layer II. In the rest 4 pairs, neurons were in layers I and II/III respectively. These results suggest that interneurons in layers I or II/III could target to both pyramidal neurons and interneurons all over the superficial layers. We also compared the properties of uIPSCs in pairs of In-Py and In-In neuron. Significant faster kinetics, such as rise time, decay time and half-width, were found for uIPSCs in interneurons than those in pyramidal neurons (Figure 3E-G). However, the latency of IPSCs was not statistically significant between two groups (P = 0.09, Figure 3D).

### Reciprocal connections between ACC neurons

**Figure 4:**
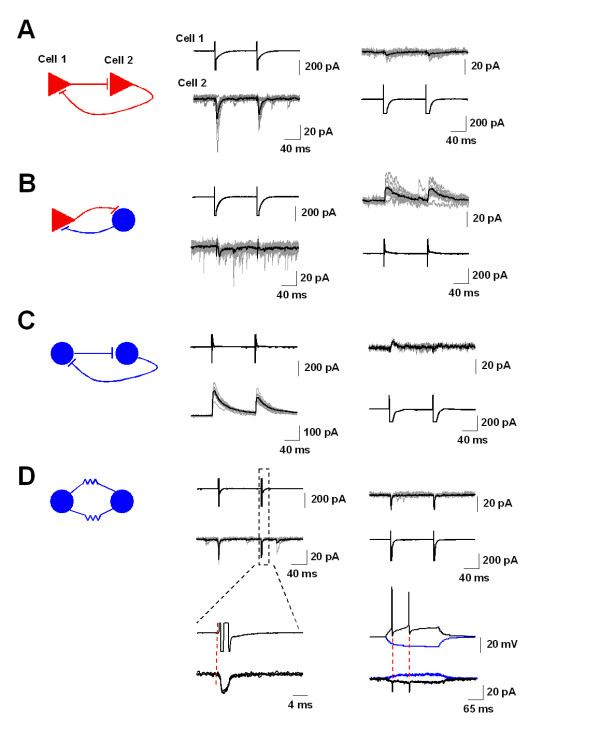
**Bidirectional connections between ACC neurons**. **(*A*) **Reciprocal connections between two pyramidal neurons. Left, diagram of the connection. Middle, two successive stimulations (1 ms, 90 mV) in cell 1 (upper) induced inward currents in cell 2 (lower). Original traces are shown in gray and the averaged trace is shown in black. Right, stimulations in cell 2 (lower) induced inward currents in cell 1 (upper). Original traces are shown in grey and the averaged trace is shown in black. **(*B*) **Diagram and sample traces of reciprocal connections between pyramidal neurons and interneurons. **(*C*) **Diagram and sample traces of reciprocal connections between two interneurons. **(*D*) **Diagram and sample traces of electrical coupling between two interneurons. The dashed rectangle is magnified and the latency of stimulation and response is indicated by red dash lines. In the right lower corner, action potentials induced in cell 1 are coupled with inward currents in cell 2.

We noticed that in functional pairs of ACC neurons, there exist reciprocal connections. For example, 2 out of 14 pairs of Py-Py show bidirectional transmission (Figure 4A). Between pyramidal neurons and interneurons, there are 8 out of 27 pairs were reciprocal (Figure 4B). Among 8 pairs of In-In, 2 pairs were bidirectional (Figure 4C), including 1 pair of electric-like coupling (Figure 4D). The electrically coupled interneurons were located in layers I and II/III respectively. There is no latency between stimulations and responses. Moreover, action potential firing in one neuron induced immediate inward current with fast kinetics of <3 ms half-width (Figure 4D)

### Morphology of neurons with functional connections in the ACC

**Figure 5:**
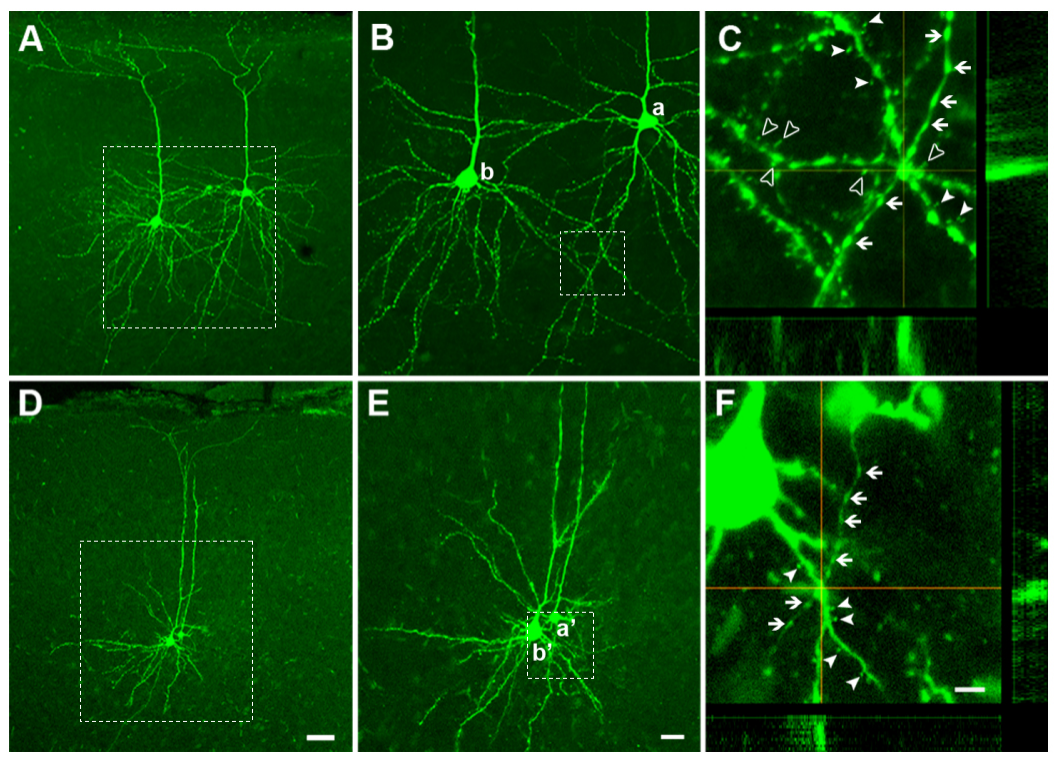
**Confocal images of biocytin-labeled ACC neurons with functional connections**. **(*A*-*C*) **Two pyramidal cells (a and b) show the possible synaptic contacts. The rectangular area in A is shown in B with a higher magnification. C is the three-dimensional view of the rectangular field in B, in which an axon from cell (a) makes close connections with two dendrites from cell (b). Arrows show the axon varicose swellings and blank and filled triangles show the dendrite spines. **(*D-F*) **One interneuron (a') makes close contact with one pyramidal cell (b'). The rectangular area in D is shown in E with a higher magnification. F is the three-dimensional view of the rectangular area in E, in which an axon from cell (a') makes close connections with one dendrite from cell (b'). Bars equal to 50 (A and D), 20 (B and E) and 5 (C and F) microns, respectively.

Biocytin was loaded into some of the recorded neurons, which was followed by immunostaining and imaging of neuronal morphology under confocal microscopy. Under confocal microscopy, all of the identified pyramidal neurons (n = 8) and interneurons (n = 6) were located in layers II and III of ACC. Pyramidal cells have bigger somata (15-30 μm) than interneurons (10-15 μm). Typically, a pyramidal cell had one main apical dendrites, which ascended from the soma, stretched into layer I and bifurcated in a tuft there. The proximal dendrites gave off many branches from the soma and arborized extensively, covering a spherical field with diameter equaled to 200-400 μm. A typical interneuron has no or short apical dendrite, less branches of proximal dendrites and smaller covering field with diameter equaled to 40-100 μm. Figure 5 shows pairs of Py-Py (Figure 5A-C) and In-Py (Figure 5D-F) neurons. By using three-dimensional analysis, we found that presynaptic boutons (with obvious varicose swellings) have very close contacts with postsynaptic spines (Figure 5C and 5F), suggesting the possible locations of synaptic contacts.

### Pharmacological identification of excitatory and inhibitory connections between ACC neurons

**Figure 6:**
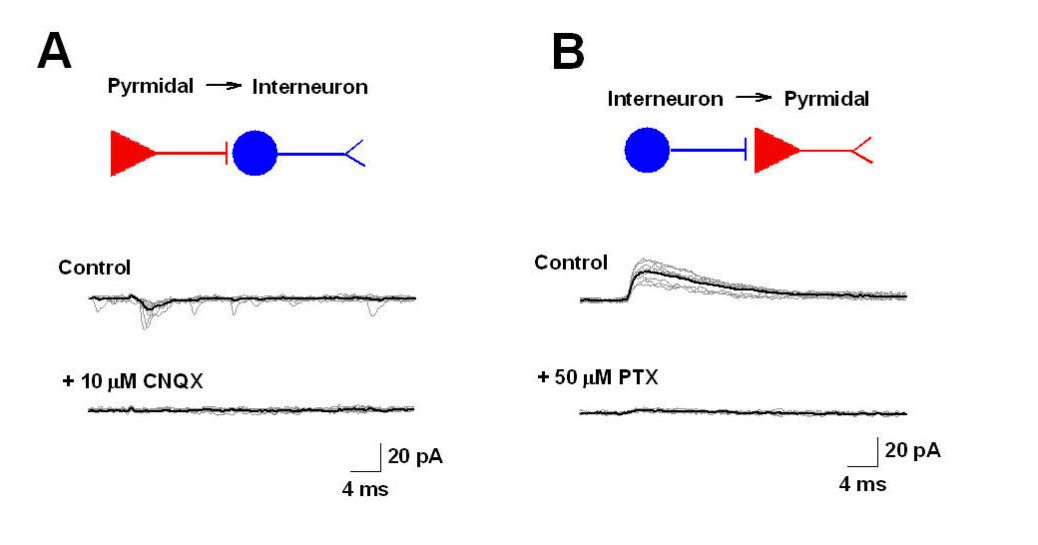
**Pharmacological studies of uEPSCs and uIPSCs**. **(*A*) **Unitary EPSCs between Py-In were inhibited by CNQX. The postsynaptic interneuron was held at --70 mV. Unitary synaptic responses were obtained by firing presynaptic pyramidal neuron. And the responses were completely abolished by bath application of CNQX. Original traces were shown in grey and the averaged trace was shown in black. **(*B*) **Unitary IPSCs between In-Py were blocked by PTX. Postsynaptic pyramidal neuron was holding at 0 mV. Unitary synaptic responses were obtained by firing presynaptic interneuron. And the responses were completely abolished by bath application of PTX. Original traces were shown in grey and the averaged trace was shown in black.

To study further the pharmacological properties of the excitatory and inhibitory connections between ACC neurons, the AMPA/kainate receptor antagonist CNQX (10 μM) and the GABA_A _receptor antagonist picrotoxin (PTX, 50 μM) were used (Figure 6). We found that CNQX could completely inhibit the synaptic responses of Py-In pairs (n = 3, Figure 6A), while PTX blocked the synaptic responses of In-Py pairs (n = 3, Figure 6B) after bath application of each antagonist for 5 minutes. These results confirm the identities of uEPSCs and uIPSCs, which are mediated by AMPA/kainate and GABA_A _receptors, respectively.

### Intracellular CI^-^-dependent IPSCs from interneurons to pyramidal neurons

**Figure 7:**
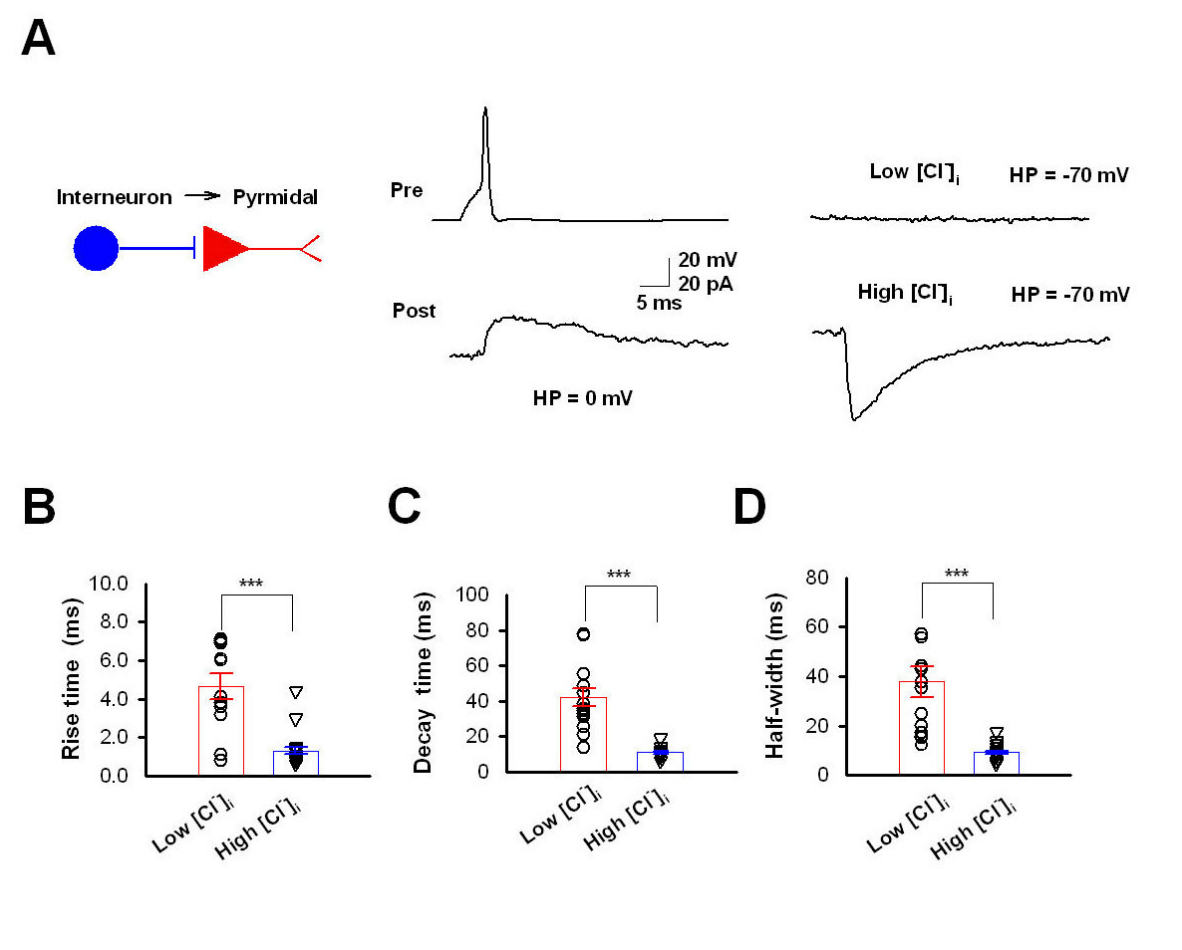
**Unitary IPSCs in ACC pyramidal neuron with high [Cl^-^]_i_**. **(*A*) **Outward and inward uIPSCs in ACC pyramidal neurons with low [Cl^-^]_i _and high [Cl^-^]_i_, respectively. Left, diagram of the connection. Middle, interneuronal firing (upper) induced outward uIPSCs in the postsynaptic pyramidal neurons at a holding potential of 0 mV with low [Cl^-^]_i _(lower). Right, no current is induced if holding at of --70 mV with low [Cl^-^]_i _(upper), while an inward current is obtained at a holding potential of --70 mV if the same neuron is re-patched with high [Cl^-^]_i _intracellular solution (upper). **(*B-D*) **Pooled data (open circle and triangle) and summarized results (bar graph) indicate the rise time ***(B)***, decay time ***(C) ***and half-width ***(D) ***of uIPSCs obtained under low [Cl^-^]_i _and high [Cl^-^]_i_. Unitary IPSCs with high [Cl^-^]_i _have faster kinetics than those with low [Cl^-^]_i_.

During development or under pathological conditions, there is a higher concentration of intracellular Cl^- ^([Cl^-^]_i_), which mediates the excitatory, instead of the typically inhibitory, effect of GABAergic responses during those states [24-27]. We wanted to see whether the polarity and properties of GABAergic transmission are also dependent on the postsynaptic Cl^- ^concentration in the ACC. To address this question, we performed dual recordings in interneuron-pyramidal neuron pairs with low [Cl^-^]_i _(7 mM) or high [Cl^-^]_i _(67 mM). In functional connections from an interneuron to a pyramidal neuron, outward current was evoked at a holding potential of 0 mV, while no current was observed at a holding potential of -70 mV with low [Cl^-^]_i_. In the same neuron, the inward current appeared at a holding potential of -70 mV after a re-patch of the postsynaptic neuron with high [Cl^-^]_i _(Figure 7A). These results confirmed that the polarity of the GABAergic response is determined by the concentration of postsynaptic Cl^-^. We successfully recorded 19 pairs of In-Py with high [Cl^-^]_i _in the postsynaptic pyramidal neurons. The kinetics of outward and inward GABAergic responses were compared and we found the inward GABAergic responses exhibited a much shorter rise time, decay time and half-width (P < 0.001, Figure 7B-D). The different kinetics could be attritubed to the different holding potentials [28] or the polarity of the GABAergic current influenced by the different [Cl^-^]_i_.

### Paired-pulse depression of synaptic connections between ACC neurons

**Figure 8:**
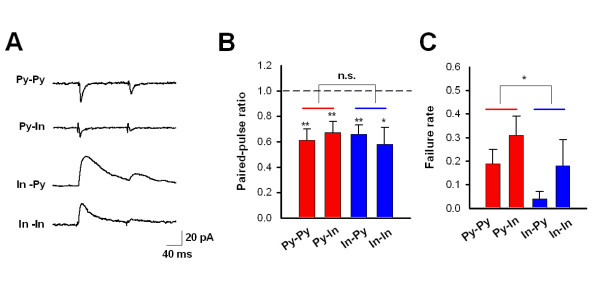
**Paired-pulse ratio and failure rate of four types of synapses in the ACC**. **(*A*) **Traces showing postsynaptic currents in the four types of synaptic connections induced by the two successive presynaptic stimulations (100 ms interval). **(*B*) **Summarized results of paired-pulse depression in all four types of synaptic connections. There is no significant difference between excitatory (pooled Py-Py and Py-In, indicated as a red line) and inhibitory (pooled In-Py and In-In, indicated as a blue line) synaptic transmission. **(*C*) **Summarized results of failure rates in all four types of synaptic connections. There is a significantly lower failure rate for inhibitory (pooled In-Py and In-In, indicated as blue line) than for excitatory (pooled Py-Py and Py-In, indicated as red line) transmission.

The paired-pulse ratio (PPR) is a commonly used parameter to indicate the presynaptic release probability [29]. Therefore, we wanted to compare the PPRs in the four types of synaptic connections described above. In contrast to what we know about excitatory transmission in the ACC [30], the PPR for uEPSC at a 100 ms interval showed significant depression rather than facilitation (for Py-Py, n = 9 out of 10 pairs, P < 0.01; for Py-In, 9 out of 12 pairs, P < 0.01, Figure 8A and 8B). There is also a significant paired-pulse depression of uIPSCs for pairs of In-Py and In-In, which is consistent with the PPR of evoked IPSCs by bulk local stimulation [22]. No difference was found for the PPR between the excitatory and inhibitory synaptic transmission (Figure 8B).

When we calculated the PPR, we ruled out the event if the first stimulation failed to induce the current. Therefore, we compared the failure rate of the four types of synaptic transmission. Noticeably, there is very low failure in the pair of In-Py, suggesting the high release probabilities of this type of synaptic connection. When we pooled the excitatory and inhibitory transmission respectively, we found that the failure rate is higher in excitatory transmission than that in inhibitory synaptic transmission (Figure 8C).

## Discussion

In the present study, we examined the intracortical connections of pyramidal neurons and/or interneurons in the superficial layers of the ACC using dual patch clamp recordings. One technical advantage of dual recording is that it avoids stimulation of passing fibers and allows investigation of local synaptic connections. Four types of synaptic pairs were characterized: (1) from pyramidal neuron to pyramidal neuron; (2) from pyramidal neuron to interneuron; (3) from interneuron to pyramidal neuron; (4) from interneuron to interneuron. In addition, there are reciprocal connections between these pairs. Interestingly, we found that the unitary postsynaptic current showed faster kinetics to interneurons than that to pyramidal neurons. However, the failure rate is higher in glutamatergic transmission than in GABAergic transmission. These results suggest that the postsynaptic neuronal type determines the kinetics of synaptic current, while the presynaptic neuronal type determines the release probability. It has been reported that different AMPA receptors are expressed in pyramidal neurons and interneurons [31]. For example, a GluR2-lacking AMPA receptor is expressed mainly in interneurons but not in pyramidal neurons in the amygdala [32]. Therefore, this may also explain the different kinetics of uEPSCs in pyramidal neurons and interneurons in the anterior cingulate cortex. Similarly, different subunit composition of GABA_A _receptors may also underlie the different kinetics of uIPSCs in pyramidal neurons and interneurons. Future experiments are needed to address the questions.

We have demonstrated the existence in the ACC of all proposed connections shown in Figure 1. For example, pyramidal neurons form synapses with pyramidal neurons and interneurons in layers II/III and also send projections to layer I interneurons; interneurons in layer I can target pyramidal neurons and interneurons in layers II/III, and also interneurons in layer I. In addition, interneurons in layers II/III can form synapses with all types of cells in the same layer or those in layer I. Since the pyramidal neurons and interneurons may also be divided into several subtypes based on their firing patterns [24,33], the present study underestimates the complexity of synaptic connections. The synaptic properties of ACC neurons need to be characterized further based on the different subtypes of pyramidal neurons and interneurons, rather than just by comparing the excitatory and inhibitory synaptic transmission or connections between pyramidal neurons and interneurons.

Short-term plasticity such as paired-pulse facilitation (PPF) and depression (PPD) is important for synaptic communication in the brain. PPF is generally explained as an increase of release probability during a second stimulus, arising from prior accumulation of residual Ca^2+ ^near active zones, while PPD is thought to reflect depletion of the pool of readily releasable vesicles or inhibition of calcium currents in the presynaptic terminal [29,34]. In the present study, we found that uIPSCs exhibit PPD, which is in agreement with our previous work using field stimulation [22] and suggest the possible high release probabilities of GABAergic synapses in the ACC. Consistently, the failure rate of inhibitory transmission is low, particularly for the pair of In-Py. However, we found that uEPSCs also show PPD in most pairs recorded, which is in contrast to the PPF of evoked EPSCs induced by field stimulations in the ACC [30,35]. Two reasons may account for the discrepancy. First, there are differences in the stimulating fibers. We only activate the presynaptic pyramidal neurons in the layer II/III in the current study, while the bulk stimulation in the layer II/III could also trigger the release from synaptic terminals originating from the medial thalamus. Strong PPF was reported for synaptic transmission from medial thalamus to layer II/III in the ACC [36]. Second, when we analyze the paired-pulse ratio, we exclude events with failure at the first stimulation. Considering around 20-30% failure rate of uEPSCs and the most likely PPF of these events, the ratio may not reflect the short-term plasticity in situ. It has also been reported that uEPSC to interneurons show different short-term plasticity dependent on postsynaptic interneuron types, such as PPD for fast-spiking neurons or multipolar cells while PPF for low-threshold spiking neuron or bitufted cells [37-39]. Although we did not identify the interneuronal types in the present study, we believe that most interneurons we recorded are fast-spiking interneurons based on the firing patterns (Figure 1B) and action potential properties (Table 1).

Neurons in layers V and VI provide the main output of the ACC. The communication between these layers and superficial layers is critical for the integration of the ACC circuit and the execution its related brain functions [40]. Therefore, future study is needed to extend the characterization of synaptic connections between deep and superficial layers in the ACC. In addition, in vitro and in vivo studies have shown plastic changes in the ACC after pathological conditions such as chronic pain [14-16,40,41]. Future experiments using dual recording could explore and uncover the synaptic mechanisms of these plastic changes at the single synapse level.

## Competing interests

The authors declare that they have no competing interests.

## Authors' contributions

LJW, XL and MR carried out electrophysiological experiments, TC performed staining experiments, LJW drafted the manuscript, MZ coordinated the study. All authors read and approved the final manuscript.
